# Abstract of the Proceedings of the Buffalo Medical Association

**Published:** 1863-04

**Authors:** J. B. Samo

**Affiliations:** Secretary pro tem.


ART. III.—*Abstract of the Proceedings of the Buffalo Medical Association.*

TUESDAY EVENING, March 3d, 1863.

Dr. T. T. Lockwood, President *pro tem.* in the Chair.

The Secretary being absent, and minutes of last meeting not being at hand, the reading was dispensed with. Dr. J. B. Samo was appointed Secretary *pro tem.*

The election of Dr. Peters was moved. Objection was made by Dr. Strong on the ground of his not having perfected his membership in the County Society, as required by the By-Laws. The objection was waived, however, and Dr. Peters was declared a member of the Association on complying with the By-Laws.

*Dr. Wyckoff* proposed Dr. Smith for membership.

The Committee on Fee Bill, through Dr. Lockwood, Chairman, reported progress, and further time was granted.

*Dr. Rochester* gave an interesting account of his examination of the bodies of the murdered woman, Mrs. Fraser and her three children.

*Dr. Rochester* reported the prevalence of roseola, a disease resembling measles in its eruption, but with milder constitutional symptoms.

*Dr. Lockwood* had seen some cases also.

*Dr. Wyckoff* spoke of a peculiar eruption on the face of an Irishman, spreading through the beard and suppurating.

*Dr. Strong* suggested, whether the disease called measles, and prevailing so extensively in our army, might not be in fact, roseola, it seeming improbable that so many should never have had measles before.

There being nothing of interest before the Association, Dr. Rochester moved to adjourn. Adjourned to first Tuesday in April.

J. B. Samo, *Secretary pro tem.*

